# An Overview of Additive Manufacturing Technologies—A Review to Technical Synthesis in Numerical Study of Selective Laser Melting

**DOI:** 10.3390/ma13173895

**Published:** 2020-09-03

**Authors:** Abbas Razavykia, Eugenio Brusa, Cristiana Delprete, Reza Yavari

**Affiliations:** 1 Department of Mechanical and Aerospace Engineering, Politecnico di Torino, 10129 Torino, Italy; eugenio.brusa@polito.it (E.B.); cristiana.delprete@polito.it (C.D.); 2 Mechanical and Materials Engineering Department, University of Nebraska-Lincoln, Lincoln, NE 68588-0526, USA; mrezayavari89@gmail.com

**Keywords:** additive manufacturing, extrusion, photopolymerization, material jetting, laminated object manufacturing, powder bed fusion, selective laser melting, melt pool, numerical simulation

## Abstract

Additive Manufacturing (AM) processes enable their deployment in broad applications from aerospace to art, design, and architecture. Part quality and performance are the main concerns during AM processes execution that the achievement of adequate characteristics can be guaranteed, considering a wide range of influencing factors, such as process parameters, material, environment, measurement, and operators training. Investigating the effects of not only the influential AM processes variables but also their interactions and coupled impacts are essential to process optimization which requires huge efforts to be made. Therefore, numerical simulation can be an effective tool that facilities the evaluation of the AM processes principles. Selective Laser Melting (SLM) is a widespread Powder Bed Fusion (PBF) AM process that due to its superior advantages, such as capability to print complex and highly customized components, which leads to an increasing attention paid by industries and academia. Temperature distribution and melt pool dynamics have paramount importance to be well simulated and correlated by part quality in terms of surface finish, induced residual stress and microstructure evolution during SLM. Summarizing numerical simulations of SLM in this survey is pointed out as one important research perspective as well as exploring the contribution of adopted approaches and practices. This review survey has been organized to give an overview of AM processes such as extrusion, photopolymerization, material jetting, laminated object manufacturing, and powder bed fusion. And in particular is targeted to discuss the conducted numerical simulation of SLM to illustrate a uniform picture of existing nonproprietary approaches to predict the heat transfer, melt pool behavior, microstructure and residual stresses analysis.

## 1. Introduction

Additive Manufacturing (AM) processes enable the transition from analog to digital manufacturing, using Computer Aided Design (CAD) software to encourage the 3D objects are being built layer-by-layer via direct material deposition onto the substrate. In contrast to the conventional subtractive and formative manufacturing processes, such as machining operations that produce the parts by material removal from a bulk, AM processes create 3D components by adding and stacking layers of material onto each other [1,2,3]. Conceptualization freedom, rapid prototyping and capability to create complex shapes and geometries are the superior AM advantages that in corollary to these, AM processes recently are widely used in aerospace [4], bio-engineering [5], automotive and product development [6,7]. AM can be helpful in two ways, either for direct prototyping in which it is used to fabricate the part, or indirect prototyping that is used to produce the tools required to be applied in conventional processes or a production assembly line such as jigs and fixtures.

Due to the nature of temperature distribution and steep thermal gradients in the part along the build direction, AM parts quality and mechanical performance are under the influence of a wide range of parameters. This rapid heat exchange affects surface quality and may lead to defects such as warping and thermal stress-induced cracking [8]. Experimental investigation of AM processes and their parameters contribution into the component quality and performance, would be expensive in terms of time and cost that due to the vast number of the variables and their interactions which should be taken into account [9].

Hence, to alleviate these challenges in studying AM processes, numerical simulation approaches have been widely used for their visibility and forward-looking value. Numerical simulation approaches would be an effective way to gain insight into the processes of physics and control [10,11]. Among the AM processes, selective laser melting (SLM) has been widespread and used for different industrial applications due to its superior capabilities to produce complex parts with adequate mechanical properties and performance [12,13]. SLM made parts generally require post processing and are seldom used as-built, therefore these result in SLM attracting industries and academia to make an effort to obtain a deep understanding of process principles [14].

The majority of the published review articles are delegated to cover experimental investigations of SLM, and technical considerations and findings. Furthermore, due to the contribution of the large number of influential parameters, a main challenge is to ensure the part quality, process repeatability and consistency. Regarding the fact that SLM is a heat transfer and repeated phase change process, therefore, it would be far more useful and trustworthy to summarize the conducted studies to numerically simulate the SLM in terms of heat transfer, melt pool dynamics and microstructure as well as induced residual stress. Hence, this review paper is organized in two sections; the first section deals with presenting an overview of AM processes and their technical considerations, and the next section is devoted to discussing and addressing the studies that made an effort to numerically simulate heat transfer, melt pool behavior, microstructure evolution and induced residual stress during SLM operation.

## 2. Additive Manufacturing Processes

**Figure 1:**
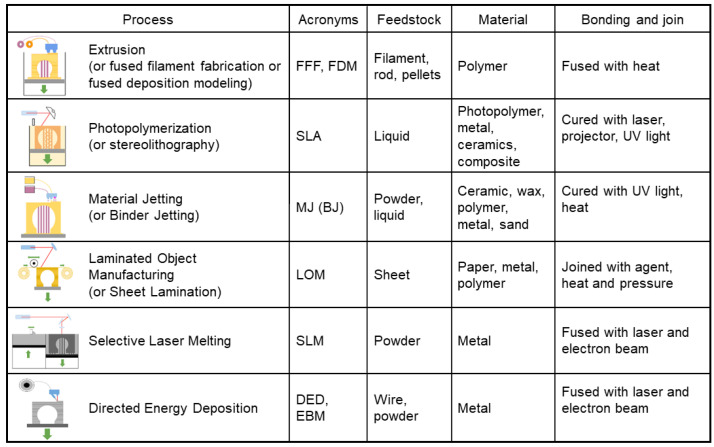
Overview scheme on additive manufacturing (AM) processes.

Additive manufacturing encompasses a wide range of technologies, materials and tools such as a vast number of software and relevant technical issues. AM processes are summarized in Figure 1 to identify the candidate process for concerned applications and materials to provide an overview of the available technologies in comparative ways [15]. Getting insight into AM process principles is essential to adequate technology to meet the part functionality criteria. Therefore, this section aims to give an overview of AM methods and to address the main operating principles.

### 2.1. Extrusion

**Figure 2:**
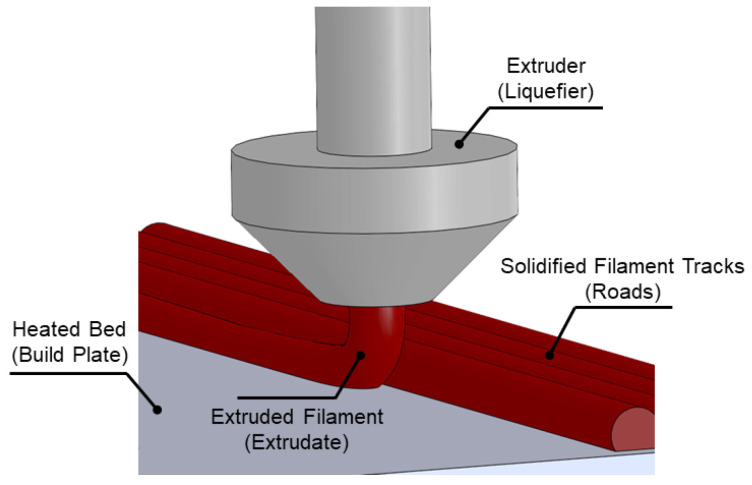
Schematic of fused filament fabrication (FFF).

Extrusion, which is typically known as Fused Filament Fabrication (FFF) or Fused Deposition Modeling (FDM), begins by slicing 3D CAD design into layers. It is a widely known and used AM process in which a nozzle extruding molten plastic builds a 3D part layer by layer, until the final geometry is obtained [16]. A broad application spectrum can be achieved from small to large, from simple to complex, from personal fabrication to a professional one such as tooling, jigs and fixture and mold production. The surface quality and final mechanical properties are under the influence of production rate (speed) and in addition extruded AM parts come with a little bit of anisotropy which means the component strength in build direction is underneath the other directions. Support structure is required that can be considered as break away or soluble, using different materials from the build materials as shown in Figure 2. In professional extrusion AM machines, the build volume is heated to relax thermal stress as the part is built, to improve dimensional accuracy. FFF or FDM is an ideal tool for fast and functional prototyping and fabrication of manufacturing aids and low-volume end-use parts using wide variety of material, ranging from thermoplastics to metal-thermoplastic composite feed-stock, for which, regarding the proposed material, further post-processing is needed to obtain the desired part and to meet the requirements [17,18]. Porosity formation and strand morphology are critical issues in extrusion that affect the part performance and are under significant influence of both the layer thickness and the strand-to-stand distance. It was reported that a lower filament layer thickness and the strand-to-strand distance led to smaller porosity and larger inter- and intra-layer bond line densities [19].

### 2.2. Photopolymerization

**Figure 3:**
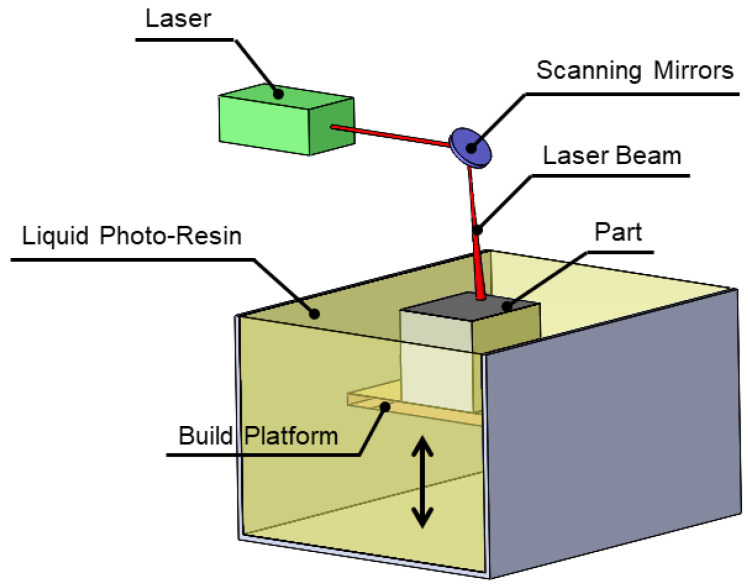
Schematic of stereolithography (SLA).

Photopolymerization, which is generally known as Stereolithography (SLA), encompasses the curing of a photosensitive monomer resin using a scanning laser or UV radiation and transferring photoresin fluid into a crosslinked solid [20]. SLA promotes the fabrication of highly detailed parts with dimensions ranging from the micrometer- to meter-scales with broad applications such as dental models, hearing aids, fast prototyping and tooling. Regarding Figure 3, part cross section over a certain depth of photoresin is scanned by laser beam via accurate movement of orthogonal turning mirrors, after accomplished the scanning, the build platform moves downward and the recoater feeds an adequate amount of the photoresin before the next layer is scanned [21]. The post curing process is of paramount importance to completing the crosslinking reaction and reaching the desired mechanical properties [22]. Mechanical properties such as strength and Young’s modulus of an SLA made component can be engineered over a wide range by tailoring the rheological behavior (viscosity) and photoresin photoreactivity [23]. In addition, photopolymer network density plays a remarkable role in governing the mechanical properties, for instance in biomedical and tissue engineering, elastomeric properties are needed, which can be achieved using photoresin with lower network density and can replace a large fraction of resin with a solvent [24].

### 2.3. Material Jetting

**Figure 4:**
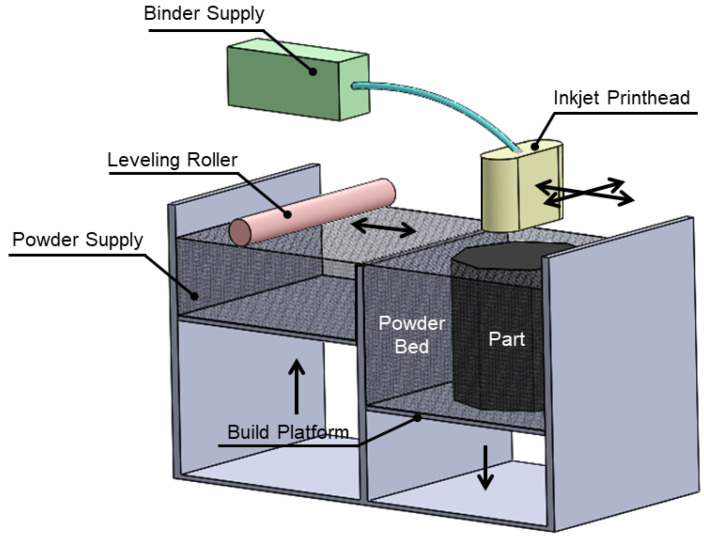
Material Jetting (MJ).

Material Jetting (MJ) is an AM method in which droplets of the liquid feed-stock is jetted through spatial control over the desired component cross section following a curing mechanism using a UV lamp as shown in Figure 4. Then, the build platform moves down to be prepared for a new layer deposition onto a previously-cured layer, this process continues to achieve the final desired part. MJ fabrication can be accomplished by applying adequate material with proper surface tension and viscosity; in corollary to this there are constrains to jet molten metals or thermal plastics. Droplet ejection from the printer head and landing control on the substrates is critical to MJ [25]. MJ is mostly used to fabricate the parts using polymers but it has capability to be utilized to print metals and ceramics. The key determinant parameter is the material capability to be deposited as droplet through the jetting print-head. Multi-Material Jetting (MMJ) through several individual nozzles, enables local specification of color and stiffness, but anisotropy might be an issue due to the nature of how the UV light is exposed, therefore it should be taken into account the deal between reaction of different material to the amount of UV energy which is depend on the surfaces’ orientations and distances from the UV source [26,27]. In general porosity and anisotropy could arise during layer-by-layer printing process which may results in imperfect or interlayer bonding weakness [28].

### 2.4. Laminated Object Manufacturing (LOM)

**Figure 5:**
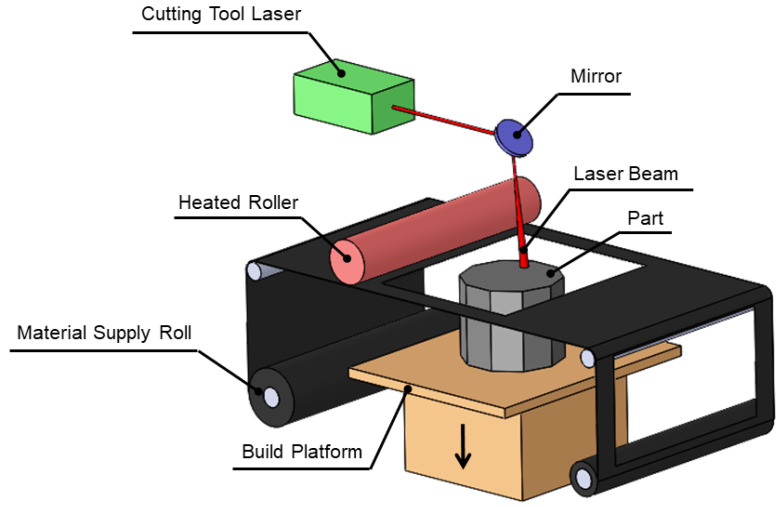
Laminated Object Manufacturing (LOM).

Laminated Object Manufacturing (LOM) is an AM process in which consecutive layers of paper sheets covered by adhesive applied to one side are continuously bonded and cut with a laser to form a 3D component as demonstrated by Figure 5. Build material is provided either on a roll or in the form of sheet-stock and heated platen or a moving roller promotes the pressure to strengthen the interlayer bonding. LOM of composite materials is enabled so that interlayer bonding is obtained through the melting and fusion of thermoplastic or thermoset polymers which infiltrate the fiber feedstock [29]. A class of LOM called Ultrasonic Additive Manufacturing (UAM) encourages hybrid fabrication of room-temperature metal deposition with CNC milling. The required pressure and force to empower the interlayer bonding is obtained using downward force and ultrasonic vibrations to provide solid-state atomic bonding to the substrate with minimal heating. Then, excess material is removed through a subtractive process (milling) to obtain the desired geometry. The main unique feature of LOM is to produce complicated 3D parts with less fabrication and a lower post processing cost [30,31].

### 2.5. Powder Bed Fusion (PBF)

**Figure 6:**
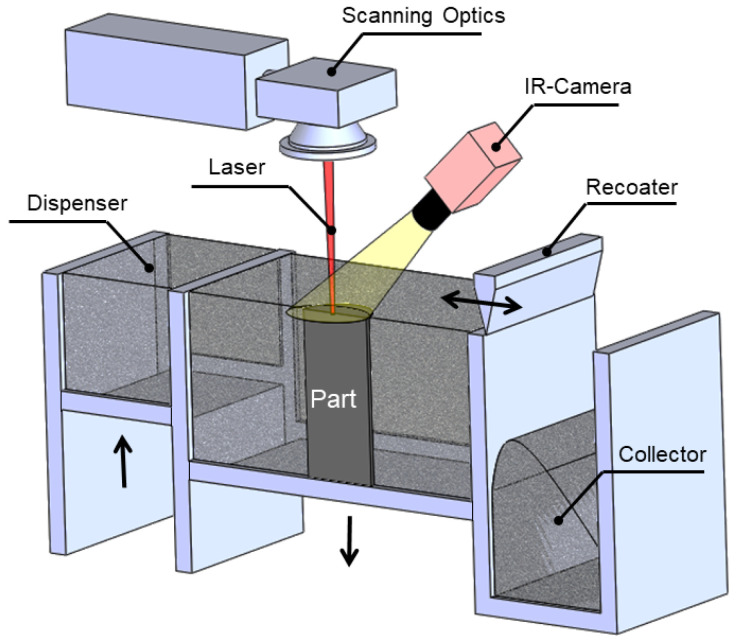
Selective laser melting (SLM).

Powder bed fusion AM technologies fabricate 3D objects by melting powders of powdered feedstock using a laser, electron beam or intense infrared lamps. Selective Laser Melting (SLM) is categorized under powder bed fusion technologies which is accomplished through consecutively melting and solidifying build material powder using a high power laser as illustrated in Figure 6. SLM is a widely used AM process due to its capability to print complex geometries with adequate mechanical properties [8,25]. Electron Beam Melting (EBM) is another class of PBF in which feedstock material is exposed to a high energy electron beam. SLM and EBM are applicable to a broad range of metals, but atmosphere control of a build chamber for the material is essential to prevent oxidation and undesired alloying. PBF of polymers is known as Selective Laser Sintering (SLS), that is, typically print thermoplastic polymers, polymer composites and in some cases ceramics. In contrast to SLM, which requires a support stricture to ease the heat transfer with the build platform, SLS does not demand the supports due to a lower temperature, as the surrounding and unfused powder provides sufficient support to the structure. In SLM and EBM, a high energy power source (laser or electron beam) scans the cross section of the part and locally melts the powders as well as partially melts the previous hatched layer to enable the interlayer bonding of the current layer with the previous one. As the laser or electron beams are shone over the powders, they may be absorbed or reflected due to the spaces and voids between the powders. There are multiple reflections of light off the powder particle surfaces as each particle acts as a spherical mirror that is not perfectly shiny, which provides some absorptivity; in addition the laser penetration is under the influence of powder packing [32]. After accomplishing the scanning of the current layer, then the recoater feeds a thin layer of powders on the top of the solidified layer and the process is repeated until the desired 3D part is achieved [33]. SLS sinters the polymer powders as the temperature is raised to the softening point of polymers. On some occasions, sacrificial materials called binder material are used in SLS to empower the sintering processes [29,34].

### 2.6. Directed Energy Deposition (DED)

**Figure 7:**
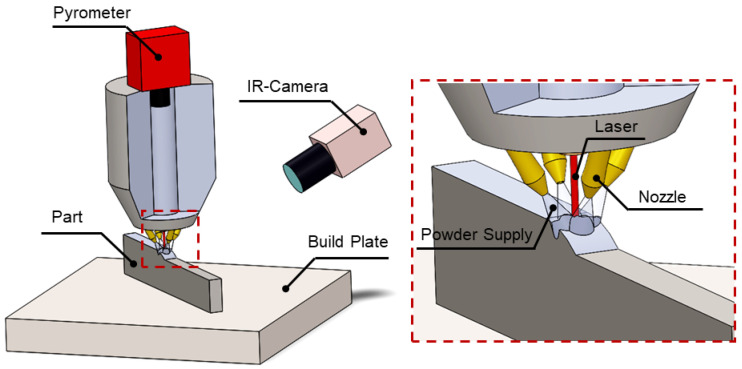
Directed Energy Deposition (DED).

In Directed Energy Deposition (DED), instead of using powder bed, build material in the form of powder or wire directly comes along with an energy intensive source such as a laser, electric arc, or electron beam to be deposited and fabricate 3D objects as shown in Figure 7. DED can be performed in two forms, powder-feed DED and wire-feed DED, the difference is the supply material system. DED has a higher flexibility and deposition rate than the PBF process as well as the encouragement of an unlimited build envelope due to the constraints of a powder bed lifting up, promoting DED to be widely used by industry [35,36]. DED is accomplished by focusing a laser beam on the building platform and simultaneously this point is bombarded by a stream of powders in which the laser beam and powders are shielded by injected inert gas to prevent oxidation and unwilling alloying. The interaction of the powder and surrounding gas flow and laser motion as well as energy source characteristics such laser density and power should accurately guarantee the melt pool formation of the desired depth. Wire-feed DED typically has a higher build rate than the powder-feed one at a lower cost. Cold spray is another type of DED in which, instead of thermal energy, kinetic energy is used to form solid state bonds between powder particles as a consequence of the high strain rate deformation of powders.

## 3. Numerical Simulation of SLM

There are so many SLM process parameters, with a huge influence on final component quality, that understanding the single effects, or their coupled interaction, is a great challenge that demands huge efforts. These variables are classified in to four groups—first, laser and scanning parameters, second, powder material properties, third, powder bed characteristics and recoating mechanisms, and the last, build volume properties. Understanding the relative importance of each SLM process is essential to guarantee the part quality. As SLM by nature is a heat transfer process, in which laser energy is penetrating the powder bed, heating and melting the powders and then the molten material is allowed to be solidified. The energy delivered to the powder bed is a function of laser power, laser mode, spot size, scanning speed and expose time [37,38].

Therefore, this section is devoted to discussing the conducted simulation of SLM to study melt pool behavior and heat transfer, surface quality, part dimensional stability, and microstructure and mechanical properties. Finite Element Method (FEM) and Computational Fluid Dynamics (CFD) are the widely applied approaches to numerically simulate SLM process to gain insight into the process physics [39,40,41,42]. The following sections give an overview of numerical approaches that are employed to simulate SLM in terms of heat exchange, melt pool formation and stability, surface quality, dimension stability and microstructure alteration.

### 3.1. Melt Pool Behavior and Heat Transfer

**Figure 8:**
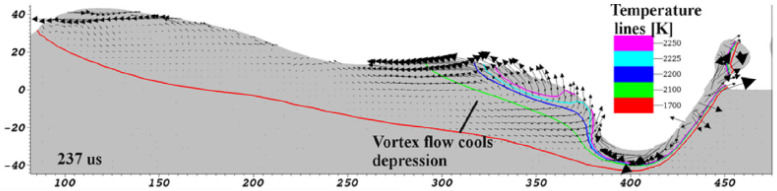
Longitudinal visualization of the laser track [43].

SLM-made part’s quality is significantly under the influence of three key parameters–laser/material interaction, heat transfer and fluid dynamics of the melt pool as well as flow of vapor and gas over the build area. Melt pool geometry and stability affects the grain growth and microstructure [32]. As the laser beam moves over the powder bed, the melt pool is elongated behind it and extended a bit in front of the laser, and due to the dependence of surface tension over the melt pool on temperature, causes the melt pool to be non uniform. In addition, the coupling effects of gas flow due to metal evaporation and surface tension result in sparks formation [43]. It is worth mentioning that cooling rate is governed by the boundary conditions, and the melting mechanism and melt pool dimension are determined by the nature of the laser and its interaction with the material. As the laser moves, the melt pool carves out behind the laser trace, and surface tension plays a remarkable role during cooling and solidification to determine the shape of the formed bead along the scanned trajectory as a laser moving on as shown in Figure 8. The surface tension is significantly under the influence of temperature which causes the scanned surface and the melt pool surface to be changing drastically which induces defects, voids and other important issues [43,44]. Due to the fact that heat transfer in SLM is a temperature dependent thermophysical phenomenon and rapid nonlinear phase transformation from solid to liquid and vice versa, the numerical simulation of melt pool behavior and heat transfer has prime importance.

Ruidi et al. employed ANSYS commercial software to study the heat transfer in SLM of Stainless Steel 316L, to understand the contribution of process parameters such as scan speed, laser power, scan interval, and scan mode. A 3D FEM was presented which comprises underneath metal substrate and upside powder that were meshed as a free grid Solid 70 and Solid90, respectively. Solid90 is a higher order version of the 3-D eight node thermal element (Solid70) which includes twenty nodes with a single degree of freedom and temperature, at each one. In the proposed FEM model heat-conduction is written as:

(1) $$\left[C_{T}\right]\left\{\dot{T}\right\}+\left[K_{T}\right]\left\{T\right\}=\left\{Q\right\},$$

where $\left[C_{T}\right]$ is the heat capacity matrix, $\left[K_{T}\right]$ is the heat conduction matrix, $\left\{\dot{T}\right\}$ and {T} are the exchange terms, nodal temperature vector and nodal temperature rate vector, respectively and {Q} is the heat flux vector. Boundary conditions in the case of heat exchange between the molten pool, ambient air, and powder bed is described by typical Fourier equations. ANSYS Parametric Design Language (APDL) has been used to incorporate the moving Gaussian heat source. It was concluded that melt pool dimension and generated thermal field are significantly under the influence of process parameters [45].

A 3D model has been proposed to numerically investigate the thermal behavior of commercially pure titanium (CPTi) powder during SLM. The transient temperature distribution T(x,y,z,t) through the domain of SLM process is governed by a 3D heat conduction equation along with adequate boundary conditions that can be expressed as [46]:

(2) $$\frac{\partial (\rho c_{p}T)}{\partial t}=\frac{\partial }{\partial x}\left(k\frac{\partial T}{\partial x}\right)+\frac{\partial }{\partial y}\left(k\frac{\partial T}{\partial y}\right)+\frac{\partial }{\partial z}\left(k\frac{\partial T}{\partial z}\right)+Q,$$

where *k* (kg/m K) is the thermal conductivity, $c_{p}$ is the specific heat capacity, ρ is the density, *t* is the time, and *Q* is the power generated per volume within the part, and initial temperature was considered to be as ambient temperature as:

(3) $$T{\left(x,y,z,t\right)}_{t=0}=T_{ambient},$$

Gaussian movement of laser has been considered that mathematically is expressed as:

(4) $$q=\frac{2AP}{\pi R^{2}}\exp\left(-\frac{2r^{2}}{R^{2}}\right),$$

where *P* is the laser power, *R* is the effective laser beam radius at which the energy density is reduced to $1/e^{2}$ at the center of the laser spot, *r* is the radial distance from a point on the powder bed surface to the center of the laser spot, and *A* is the laser energy absorptivity of the powder affected by the laser wavelength and the surface conditions and physical properties of the powder. Numerical simulation has been conducted on ANSYS Multiphysics FEM considering powder bed to be continuous and homogeneous medium. It was highlighted that the depth and width of the melt pool decrease as scan speed increases while increasing with the laser power increment.

Equations (2) and (4) are widely used to govern the spatial and temporal temperature distributions and heat source movement in SLM numerical simulation [47,48,49,50]. The coupled interaction of preheated powder particles and direct absorption of the laser energy by growing layers successfully can be represented by an adequate Gaussian energy density distribution over a surface or volume or both [51,52,53,54].

Manvatkar et al. [55] developed a three-dimensional heat transfer and melt flow model of SLM examining the effects time dependent variation, cooling rate and peak temperature. The model is relied on conservation equations for mass, momentum, and energy. The temperature of powder and thermal properties are assigned to be equal as inert gas and the initial preheat temperature. The amount of laser power that could be absorbed by the depositing surface was expressed by:

(5) $$P_{s}=\eta_{l}\left(1-\eta_{P}\right)P,$$

where $\eta_{P}$ is the fraction of the laser power absorbed by the powder in-flight, $\eta_{l}$ is the fraction of available laser power absorbed by the growing layer, and *P* is the laser power [56].

A 3D Finite Volume Method (FVM) has been proposed to simulate heat transfer and densification mechanism of Wc/Cu composite powder subjected to SLM. Surface tension due to transition from powders to solid and rapid temperature changes has been taken into consideration as well as Gaussian movement of the laser. Governing equations include mass, momentum and energy formulas to evaluate impact of the applied linear energy density (LED) on the heat exchange, melt pool dimensions, and amount of induced porosity in terms of gaseous bubbles and final densification. ANSYS Fluent software has been used with consideration of certain penetration depths of laser radiation into the powder layer, melt pool formation under the laser beam and melt infiltration into powder bed by capillary and gravitational forces. Some authors, presented comprehensive model considering both thermo-capillary force and recoil pressure induced by the material evaporation that are the forces to encourage the melt flow [57,58,59].

Considering the quasi-steady state of the melt pool affects its geometric stability, which is correlated with SLM produced part microstructure and properties. Melt pool size and shape is significantly under the influence of residual heat during raster scanning process. The residual heat would increase the melt pool size as the laser beam travels on the subsequent scanning path in which the incompletely dissipated heat from previous scanned path increases the melt pool temperature. After certain number of raster path, the melt pool size does not be affected by residual heat come. Under high laser power and low scan speed, considering longer scanning path might be required for melt pool to be fully developed [34,60,61].

Continuity, momentum and energy equations (Equations (6)–(8), respectively) have been applied to develop a 3D numerical model to examine the laser–powder–atmosphere interaction under different combination of SLM process parameters. The proposed model is enabled to study the heat transfer and melt pool dynamics taking into account the active roles of laser characteristics, temperature distribution, the powder layer properties, the nature and pressure of the environment gas, and other process parameters.

(6) $$\frac{\partial \rho }{\partial t}+\nabla \left(\rho \vec{v}\right)=0$$

(7) $$\frac{\partial (\rho \vec{v})}{\partial t}+\nabla \left(\rho \vec{v}\vec{v}\right)=-\nabla p+\nabla \left(\mu \nabla \vec{v}\right)+\rho \vec{g}+S_{m}$$

(8) $$\frac{\partial (\rho C_{p}T)}{\partial t}+\nabla \left(\rho \vec{v}C_{p}T\right)=\nabla \left(k\nabla T\right)+S_{h},$$

where *v*, *p*, μ, *g*, $S_{m}$ and $S_{h}$ are the mixture velocity vector, the pressure, the viscosity, the gravity acceleration and the source terms, respectively. It was concluded that the high pressure build chamber would reduce the convective movements in the atmosphere and evaporation of the surface [62]. Four kinds of body forces in momentum equation (Equation (7)) are incorporated and $S_{m}$ can be expressed as:

(9) $$S_{m}=\vec{S_{dam}}+\vec{f_{sur}}+\vec{f_{mag}}+\vec{p_{rec}},$$

where $\vec{S_{dam}}$ is the Darcy force responsible for dampening the velocity to zero when the temperature drops under the melting temperature [63], $\vec{f_{sur}}$ is the one of the surface tension components which is normal to the gas-liquid interface, $\vec{f_{mag}}$ represents the tangential component of the surface tension so called Marangoni forces [64], and recoil pressure $\vec{p_{rec}}$ which is caused by the evaporation [65] can be expressed as:

(10) $$p_{rec}=p_{0}\exp\left[\frac{m_{v}L_{v}}{\sigma }\left(\frac{1}{T_{b}}-\frac{1}{T}\right)\right],$$

where $p_{0}$, $m_{v}$, σ, $L_{v}$ and $T_{b}$ are the operation pressure, the molecular mass, the boltzmann constant, the latent heat of evaporation and the ambient temperature, respectively. $S_{h}$ in the energy conservation equation (Equation (8)) comprises the absorption and releasing of the latent heat of melting [66] that can be expressed as:

(11) $$S_{h}=-\rho \left[\frac{\partial }{\partial t}\Delta H+\nabla \left(\vec{v}\Delta H\right)\right],$$

where ΔH is the latent heat of the phase transformation [67].

Loong et al. [68] presented a thermal model to study powder-to-solid transition, shrinkage and vaporization taking into account the effects of laser power, scan speed and laser spot size; along this parametric FE software, COMSOL Multiphysics™ is applied to conduct the simulation.

FEM simulations have been performed to examine the impacts of laser optical penetration depth and Linear Energy Density (LED) heat transfer and melt pool size alteration [69,70,71,72]. In SLM, LED is expressed as the ratio of laser power to scan speed (LED=P/v), which governs the temperature distribution and melt pool dimension [73].

**Figure 9:**
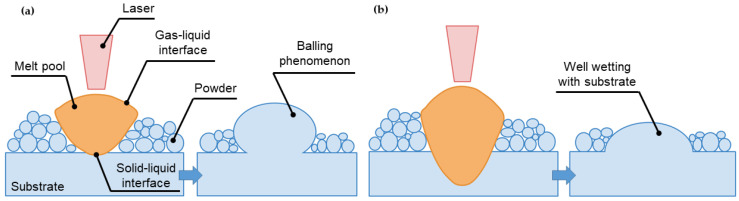
The solidification phenomenon during SLM: (**a**) partially penetration of laser into powder bed, (**b**) adequate laser penetration into powder layer.

As a consequence of not well-defined laser power and scan speed or on the other hand, inadequate LED ratio, laser unable to completely penetrates through the powders. In this circumstance, gas-liquid interface plays significant role to generate the interfacial tension which results in the molten metal tends to the spherical agglomeration with the smallest Gibbs surface free energy, as demonstrated by Figure 9a. In contrary, under adequate LED, the laser penetrates the powder totally in which melts the surrounded powders to form the melt pool and remelts the previous hatched layer that encourages the well wetting with substrate as shown in Figure 9b [74].

The generated temperature field, gradient, and the thermo-capillary convection intensity in the melt pool were considered to simulate the physical property diversity in both sides of the laser scan track. The presented numerical model enables the study of the morphology of the overhanging surface [75]. The computational frameworks have been proposed to discuses heat transfer formulation and metal deposition processes in SLM using FEM models to assess the influence of process parameters [76,77,78,79]. FEM and mathematical formulation have been developed using deal.II which is an open source finite element library to evaluate phase transformation and melt pool geometry alteration [80,81]. Along with 3D FEM, the Volume of Fluid (VOF) method was employed to track and reconstruct the free surface of the molten pool during SLM to consider volume shrinkage and temperature-dependent thermophysical parameters. It was highlighted that melt pool and geometrical instability, and continuity boundaries are significantly under influence of laser energy density [82]. The VOF method is widely used numerical technique to model the free surface or fluid-fluid interface and to track the position and shape of the molten pool surface by solving a scalar transportation equation for the volume fraction of fluid in a cell (F) as:

(12) $$\frac{\partial F}{\partial t}+\nabla \left(\vec{v}F\right)=0,$$

A cell is void when F=0, and is completely occupied by the fluid when F=1. When the value of *F* is between 0 and 1, an interface between the fluid and void exists in the cell [43,83,84,85]. Applying the VOF method enables the keyhole boundary tracking and numerically simulation as well as phase transformation and heat exchange analysis [86,87,88,89].

**Figure 10:**
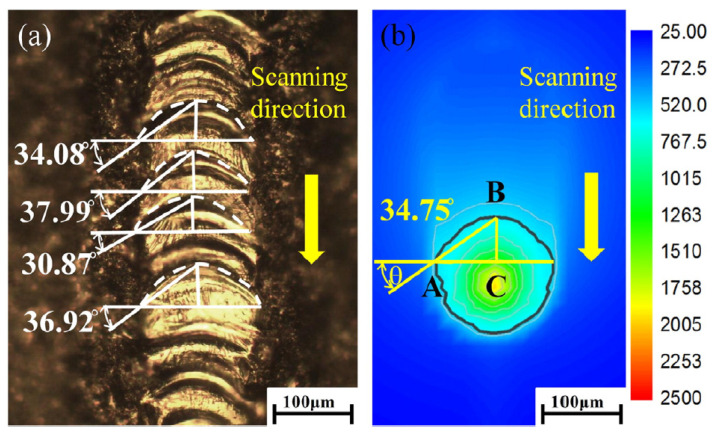
Ripple angle as indicator to examine track surface (**a**) experimental sample, (**b**) numerical simulation [90].

Track stability and ripple angle can be used as indicators to examine melt dimension and surface morphology as their coupled effects affect the SLM made components [90,91]. Zhang et al. developed a 3D FEM model to evaluate melt pool dimensions and surface characteristics. The proposed model was validated by track stability and ripple angle [92]. The ripple angle, θ, is defined as the shape of the isotherm curves as shown in Figure 10.

Height Function (HF) is broadly applied to calculate the interface curvature from VOF fractions due to its second-order accuracy and easy implementation [93,94]. Some researcher combine HF by CFD modeling, in particular with FVM to improve the solution of surface tension forces [95,96]. A HF-Lattice Boltzmann method (HF-LBM) coupled model was developed to enjoy the consideration of both computational efficiency and the important physics. The model enables the evaluation of melt pool dynamics taking into account interracial forces via surface tension, Marangoni convection and recoil pressure [97].

The influences of positive and negative defocus, Volumetric Energy Density (VED) and normalized enthalpy on the melt pool depth and stability have been examined [98]. VED is a synthetic index with physical meaning correlating laser power, hatch space, scan speed and layer thickness [99] as:

(13) $$VED=\frac{P}{hvl_{t}},$$

where *h* is the hatch space, and $l_{t}$ the powder layer thickness. However, VED is not an effective tool to completely model the physics of the melt pool [100].

**Figure 11:**
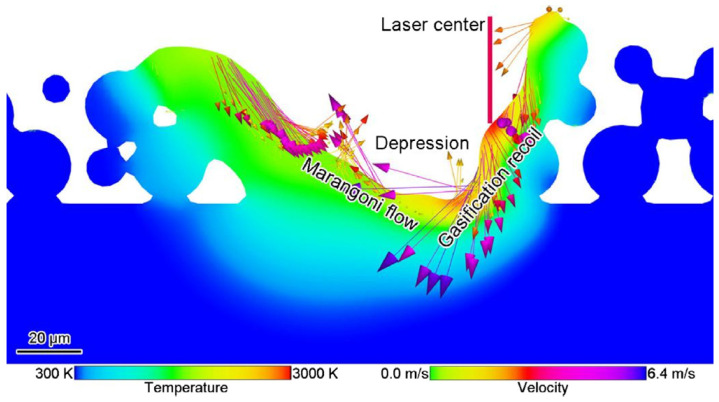
Melt pool dynamics comprising Marangoni effect, gasification recoil [102].

Theoretically, the investigation of melt pool behavior in SLM can be performed in two ways, either based on work-piece scale or on particle size. In work-piece scale, the powder layer is treated as a special material in which temperature distribution evaluation is done by setting an equivalent physical parameters and flow behavior models [101]. The so called particle scale modeling relies on the actual particle morphology in which interaction of the laser and metal particles can be calculated directly with capability to describe the melt flow through the particles [89]. Cao [102] developed a particle scale model to examine the melt pool dynamics taking into account the effects of thermodynamic factors such as Marangoni effect, gasification recoil, and gasification heat dissipation under consideration of Gaussian heat source as shown in Figure 11.

A novel approach based on spectral graph theory has been introduced to simulate heat exchange. Two benchmark heat transfer problems with planar boundaries, associated with analytical solution, 1D Finite Difference Method (FDM) and 3D FEM, have been applied to determine the precision of temperature prediction [8,103,104]. The applied approach aimed to estimate the effect of component geometry and influential parameters on instantaneous spatiotemporal distribution of temperature.

Combining the Discrete Element Method (DEM) and FVM model relying on mesoscopic scale has been applied to gain deep understanding of the powder size impact on the powder flow behavior and the resultant melting/solidification characteristics during SLM of WC/Inconel 718 composite [105]. DEM would be considered a reliable tool to describe the behavior of discrete granular materials following a given force-displacement relationship during the initial powder paving which was recently used to study the packing state of powders during the recoating in PBF [106,107,108].

An analytical model has been presented to evaluate the impact of pulsed and continuous laser emissions, considering the main spatial and temporal parameters affecting the energy delivering efficiency to the powder bed during the SLM. Process efficiency is assessed by identifying changes corresponding to duty cycle [109]. To incorporate the generated temperature field by a pulsed wave Gaussian, the solution proposed by Ravi Vishnu et al. [110] has been applied.

### 3.2. Surface Quality, Part Geometrical Stability and Residual Stresses

A comprehensive understanding of microstructure alteration due to rapid heat exchange, melting and solidification during SLM is essential to controlling the process and obtaining the desired mechanical properties and to grant the parts performance. Therefore, this section discusses and summarizes the efforts that have been made to simulate and predict the interaction of SLM parameters and components quality. FEM is widely applied to examine the impact of processes parameters to evaluate melt pool dynamics, induced residual stresses and microstructure alteration [111,112,113,114]. Thermal simulation, which is mainly fed as an input for the mechanical analysis, is also applied to assess the melt pool geometry and stability and microstructure variation along the build direction [115,116,117].

FVM has been employed to consider powder to solid transition to study heat transfer, and the effects of laser power on surface quality and balling phenomenon [118].

A computational FEM framework comprised of grains and melt pool dynamics was presented to model the mechanical response of 316 stainless steel during the execution of SLM. The proposed model examines different combinations of process parameters to obtain various melt pool size and grains based on the fact that in experiments, changing laser characteristics results in melt pool dimension alteration. The Cohesive Zone Model (CZM) was applied to describe the interaction between the melt pool boundaries in the FEM [119]. CZM is a continuous incident in which separation starts across an extended crack or cohesive zone that is limited by cohesive tractions [120].

Cellular Automata (CA) has been used to simulate dendrite angle, grain size and shape which requires prerequisites thermal simulation and determination of melt pool dimension. A coupled model comprising CA–FEM has been introduced to predict the microstructure alteration during SLM of AA-2024 feedstock. The proposed approach enables the calculation of heat transfer and grain growth, taking into account powder-to-liquid-to-solid transformation [121]. As CFD analysis can result in more accurate thermal simulation and study of the melt-pool dimension, therefore CFD was coupled with CA to predict the microstructure of Ti-6Al-4V subjected to SLM with a single track [122].

**Figure 12:**
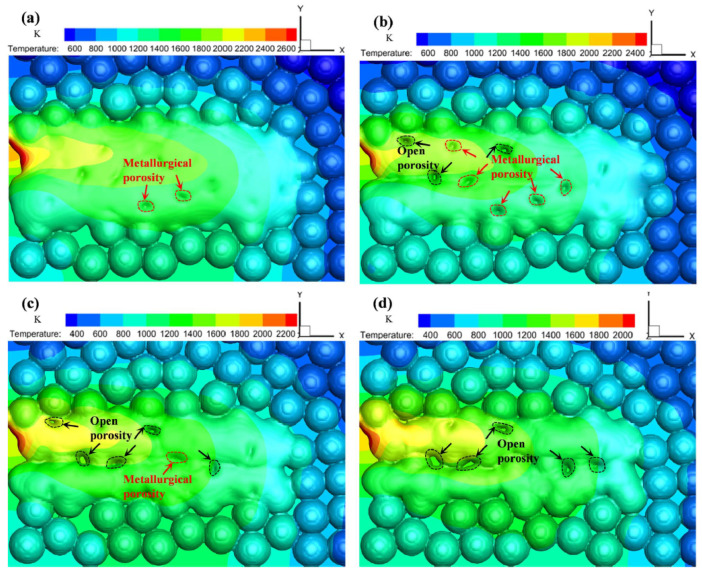
Evolution of calculated porosity on the top surface at various scanning speeds: (**a**) 200 mm/s; (**b**) 300 mm/s; (**c**) 400 mm/s; (**d**) 500 mm/s [67].

Xia et al. [67] made an attempt to evaluate the mechanism of induced porosity presenting a transient mesoscale model with a randomly-packed powder bed. The higher the scan speed, the higher rate the transformation from metallurgical porosity to open porosity, as the dissolved gasses would not space the melt due to short lifespan of melt pool as shown in Figure 12.

Wu et al. [123] introduced an FEM model using ABAQUS software to examine generated temperature and residual stress fields during SLM of AlSi10Mg. The first laser movement has been incorporated to be exposed onto the powder bed, then heat transfer was used to describe the local temperature field, and finally the solid mechanical model incorporated the derived temperature results to evaluate the imposed residual stress. Temperature-dependent material properties have been combined with phase change from powder–liquid–solid to evaluate imposed residual stress during SLM of Ti6Al4V using the ABAQUS USDFLD subroutine [124].

The influence of LED geometry stability, microstructure and micro-mechanical properties of Al${}_{2}$O${}_{3}$ have been assessed to gain insight into the solidification mechanism and thermal behavior of the melt pool applying FVM [125]. The same VED was applied to study the effect of laser power and correlate the induced residual stress with the melt pool dimension [126].

An effort has been made to examine the effects of Solid-State Phase Transformation (SSPT) and powder–liquid–solid transition on the residual stress evolution during the SLM process of Ti6Al4V. Powder–liquid–solid transition comprises melting, vaporization, solidification, shrinkage and cooling phenomena. Stress fields have been assessed via the elasto-plastic constitutive relationship including thermal strain and volumetric change strain [127].

Dong et al. [128] introduced an FEM model to simulate thermal behavior during SLM of AlSi10Mg to correlate microstructure heterogeneities, geometric accuracy, pore defects, and build orientation by solving the heat conduction equation (Equation (2)). The effective thermal conductivity of the material was considered with respect to its phase state (solid or liquid phases) as a function of porosity, thermal conductivity of metal, and surrounding gas. As the presence of structural defects, due to solidification mechanism, melt pool thermal behavior, and steep thermal gradient along the build direction as well as powder-liquid-solid thermal interactions, is irrefutable, probabilistic modeling of SLM made components might be a great tool. Ai et al. [129] introduced a probabilistic model to examine fatigue life due to manufacturing defects during casting. Due to the thermal interaction of the liquid–solid phase in casting processes, the proposed approach can be adopted to incorporate other important aspects in SLM to study the role of the induced defect, while the part is printing, on the final part performance.

**Figure 13:**
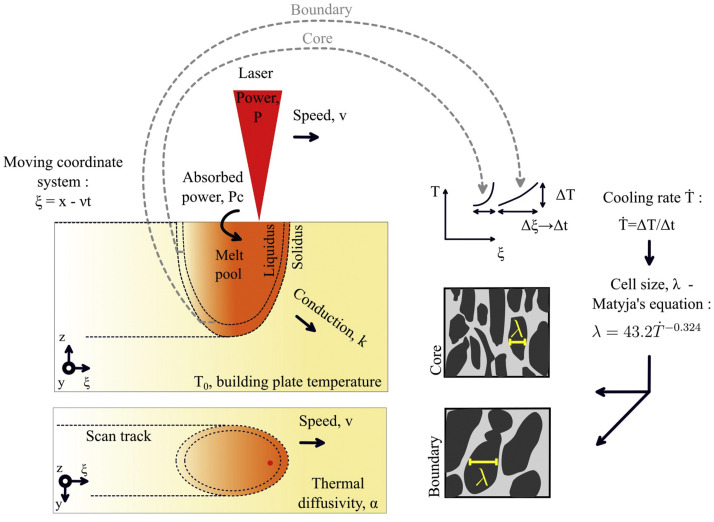
Method to compute the cell size with the melt pool depth using Rosenthal’s [130].

The relationship between the processing parameters and the size of the Heat Affected Zone (HAZ) has been studied, relying on thermal history using Rosenthal’s equation [130]. Thermal history–Rosenthal’s equation can be expressed by Equation (14), the computation methodology and variable descriptions are shown in Figure 13 [131,132].

(14) $$T=T_{0}+\frac{Pc}{2\pi Rk}e^{\frac{-v(\xi +R)}{2\alpha }},$$

where *T* and $T_{0}$ are the temperature and the building plate temperature, respectively, *P* the laser power, *c* the absorbed power coefficient, *k* the conduction, *v* the speed, α the thermal diffusivity, *R* the effective laser beam radius and ξ is the moving coordinate system:

(15) $$R={\left(\xi^{2}+y^{2}+z^{2}\right)}^{\frac{1}{2}}$$

(16) ξ=x−vt,

where *x*, *y* and *z* are the direction of the laser motion and *t* the time.

The correlation between the process parameters and obtained microstructure for individual laser tracks on Inconel 625 bare substrates has been studied. The proposed model was implemented in three levels–first, heat conduction was solved via the thermal diffusion equation, then the thermal–fluid model takes into account liquid flow considering Marangoni and next, the thermal–fluid–vaporization model takes into account the heat loss caused by vaporization. Regarding the results obtained from the thermal model, the primary dendrite arm spacing can be predicted using the Kurz-Fisher (KF) model [133].

A Phase Model (PF) has been presented to study the microstructural evolution during SLM of Inconel 718. Phase field equations coupled with the solute diffusion equation was employed to govern the phase field behavior within the domain [134]. The PF formulation was derived based on a thin interface analysis to predict dendritic formation [135]. Fallah et al. [136] presented an FEM–PF combined model to simulate directional solidification under local steady state conditions in two steps with first, the thermal analysis and then PF formulation.

## 4. Conclusions

This review survey first aimed to give an overview of Additive Manufacturing (AM) processes and in principle is targeted to summarize the previous works devoted to numerical approaches to studying Selective Laser Melting (SLM). The conducted investigations of SLM technology are mainly devoted to studying the effects of process variables such as scanning strategies, post–processing and prerequisites preparation, as well as substrate preheating, which vary from case to case.

Due to the presence of a vast number of significant variables, affecting the part quality, heat transfer, temporal microstructure and residual stress, experimental investigations would be costly and time consuming. Numerical simulation of SLM would be an effective tool to get insight into the process physics and working principles. Thermal behavior and melt pool molding are essential to governing and controlling the part quality, induced stress and mechanical properties. The majority of conducted simulations of the SLM process are mesh-based methods and, in some cases, coupled models including the Finite Element Method (FEM), the Discrete Element Method (DEM), the Finite Difference Method (FDM) and the Finite Volume Method, which are widely utilized to solve boundary, initial and eigenvalue problems. It is highlighted that the inter layer bounding is drastically affected by the melt pool dimensions and shape. Scanning speed, laser power or Linear Energy Density (LED) are the critical variables to determine the melt pool dimension, length and width. FEM is mostly applied to modelling multiple tracks and layers and in contrast, Computational Fluid Dynamics (CFD) is employed to simulate the limited number of tracks.

Thermal molding is served as a prerequisite step to analyzing the microstructure evolution such as phase transformation and dendrite arm spacing. FEM is coupled with Phase Field (PF) modeling to simulate microstructural alteration along the build direction and dendritic formation on a micro scale. PF modeling can assess how new material can be characterized to be used by SLM. CFD, due to its capability to accurately calculate the heat transfer and temperature gradient, would result in a better understanding of microstructure evolution. It is believed that this review article can furnish the solution of some issues and important aspects during the numerical simulation of SLM.
